# In Situ Stress Tensor Determination during Phase Transformation of a Metal Matrix Composite by High-Energy X-ray Diffraction

**DOI:** 10.3390/ma11081415

**Published:** 2018-08-12

**Authors:** Guillaume Geandier, Lilian Vautrot, Benoît Denand, Sabine Denis

**Affiliations:** 1 Institut Jean Lamour, UMR 7198, CNRS—Université de Lorraine, Campus ARTEM, 2 allée André Guinier BP 50840, 54011 Nancy CEDEX, France; lilianvautrot@gmail.com (L.V.); benoit.denand@univ-lorraine.fr (B.D.); sabine.denis@univ-lorraine.fr (S.D.); 2 Laboratory of Excellence on Design of Alloy Metals for low-mAss Structures (DAMAS), Université de Lorraine, 54000 Nancy-Metz, France

**Keywords:** metal matrix composite, in situ X-ray diffraction, internal stresses, phase transformation, microstructure

## Abstract

In situ high-energy X-ray diffraction using a synchrotron source performed on a steel metal matrix composite reinforced by TiC allows the evolutions of internal stresses during cooling to be followed thanks to the development of a new original experimental device (a transportable radiation furnace with controlled rotation of the specimen). Using the device on a high-energy beamline during in situ thermal treatment, we were able to extract the evolution of the stress tensor components in all phases: austenite, TiC, and even during the martensitic phase transformation of the matrix.

## 1. Introduction

Mass reduction, such as in transportation applications where it is desired to reduce fuel consumption and pollution, can be obtained by using new lighter materials with mechanical properties that are at least equivalent to those of the previous ones. This goal can be reached using Metal Matrix Composites (MMCs) reinforced with ceramic particles. In our study, a steel matrix composite reinforced with TiC particles obtained by powder metallurgy (a mixture of 75% steel powder and 25% TiC powder) allows the density of this composite to be reduced to 7 g/cm^3^, compared to 7.8 g/cm^3^ for steel alone (i.e., a decrease of 11.4% in mass). The final properties of an MMC depend on the chemical composition, on the nature of the interfaces, on the microstructure of the matrix, and on the stresses in the reinforcements and in the matrix. Many studies deal with load transfer between the phases in composite materials induced by external loading [1,2]. However, large stresses can be generated during the heat treatment, resulting from the differences in the coefficients of thermal expansion between matrix and reinforcements [3,4,5], and also from the phase transformations [6,7]. The residual stress levels and distributions are a key factor for the final properties of the MMC [8]. In a previous study [9], the evolution of the matrix and the reinforcements were analysed during the heat treatment using in situ high-energy X-ray diffraction focusing on the structural aspects. In this paper, we focus on the internal stress analysis. First, we describe the dedicated original device and the methodology that was developed to allow an in situ determination of the evolutions of internal stresses in a steel matrix composite during cooling. Then, internal stresses in the phases are analysed, emphasising the role of martensitic transformation. Finally, a 3D finite element micromechanical model is used to better understand this role, and a comparison with experimental results is discussed.

## 2. Material and Thermal Cycle

**Figure 1:**
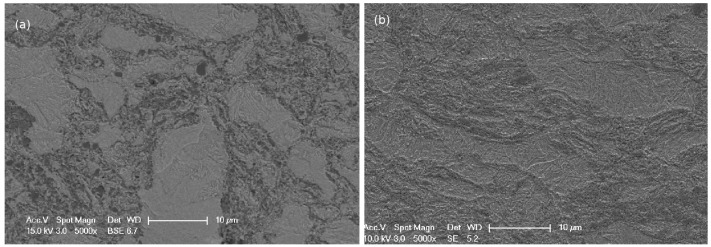
Metal matrix composite (MMC) microstructure (**a**) at initial state and (**b**) after the thermal treatment.

A metal matrix composite was elaborated using powder metallurgy by Mecachrome (France). Steel powder (32CrMoV13, see Table 1 for the chemical composition of the matrix) at a volume fraction of 75% and TiC powder at a volume fraction of 25% were milled under Argon atmosphere. The powders were consolidated by hot isostatic pressing (HIP) under a stress of 100 MPa at 1120 °C during 4 h. After hot isostatic pressing, the microstructure of the steel matrix composite (see Figure 1a) was non-homogeneous and presented two different areas: a steel area (pearlitic microstructure) without TiC particles, called the unreinforced area (lighter area), and a darker area comprising a mixture of TiC particles and steel.

**Figure 2:**
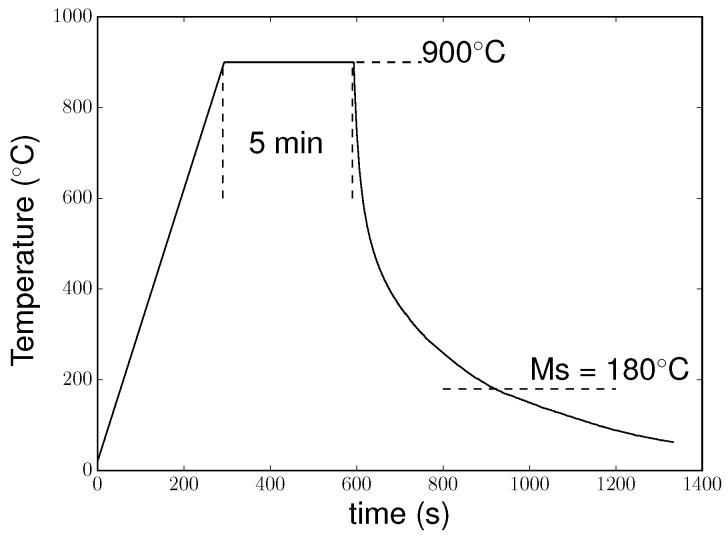
Thermal cycle applied to the MMC.

More details on the microstructure and chemical composition can be found in [9]. Figure 2 presents the thermal cycle measured by a thermocouple welded to the sample. The sample was heated to 900 °C at 3 °C·s^−1^. The temperature was held for 5 min at 900 °C, and then the sample was cooled down. To achieve cooling, the lamps of the furnace were switched off at the end of the dwell, and a controlled gas flow (argon) allowed the cooling rate to be controlled. The cooling rate was fast enough to avoid ferrite or bainite formation before the martensitic transformation at M_*S*_ temperature (measured at 180 °C). The microstructure after the thermal treatment is presented in Figure 1b: the unreinforced areas composed of martensite and retained austenite (as we show later in the paper) and the reinforced areas where the matrix was mixed with TiC particles.

## 3. High-Energy X-ray Diffraction

### 3.1. Experimental Setup

The high-energy X-ray diffraction (HEXRD) experiments were performed at the European Synchrotron Radiation Facility (ESRF, Grenoble, France) on the ID15B beam line. The in situ measurements were conducted with an 87 keV monochromatic beam. The high-energy beam allowed us to analyse a large volume of the sample (due to the low absorption of the sample), thus being representative of the bulk and lessening the surface effect. The transmitted signal was collected by a large-area 2D detector (Perkin Elmer XRD1621) that recorded the whole Debye–Scherrer rings with a maximum 2θ angle of 12°. The high photon flux gives a high-quality diffraction signal, and thus HEXRD frames can be recorded at a high frame rate (up to 10 fps). Using this method, we could follow the evolution of Debye–Scherrer rings during a thermal treatment. For our experiments, the sample was a cylinder of 3 mm diameter and 30 mm length, and the beam size was 0.4 × 0.4 mm^2^.

The sample was heated with a radiation furnace that was specially developed to perform a thermal treatment with a controlled rotation of the sample [10]. Temperature was controlled by a thermocouple welded on the sample surface, placed just below the beam path to avoid diffraction signal from the thermocouple. The sample and furnace configuration ensured that the temperature was homogeneous around the thermocouple and beam path. The rotation of the sample around its vertical axis was controlled by stopping it at fixed positions (0°, 90°, 180°, and 270°) for 0.3 s. The change between each position took 0.2 s. The sample rotation during in situ cooling and the fast acquisition rate allowed the data to be collected for determination of the full-stress tensor within one second, as we obtained all necessary orientations of the sample by combining the rotation of the sample and 2D diffraction in the perpendicular plane within two rotation steps.

### 3.2. Phase Analysis

**Figure 3:**
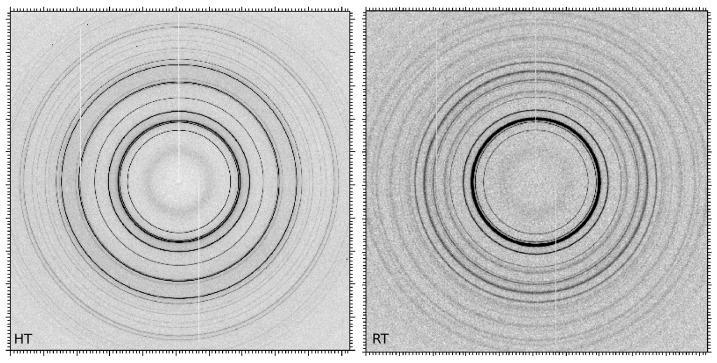

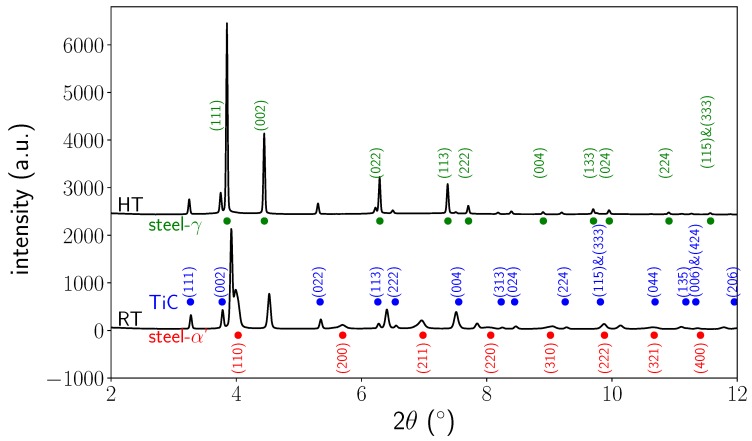
2D patterns showing austenite and TiC diffraction signals at high temperature (HT) and showing austenite, martensite and TiC diffraction signals at room temperature (RT). Diffractograms from integrated images for the different phases (austenite, martensite (α’), TiC).

As the sample presented small grains and no texture during the whole thermal treatment (see some examples of 2D patterns recorded at high temperature and at room temperature in Figure 3), a Rietveld analysis was conducted to extract phase fractions and mean cell parameters from the HEXRD frames using FullProf software [11,12]. Data were corrected (dark field and flat field) and reduced to (2θ, intensity) patterns using fit2D software [13]. Integration of the intensities was performed all around the rings.

## 4. Internal Stress Determination

From the same corrected frames used for Rietveld analysis, a stress analysis was performed by applying the $sin^{2}\Psi$ method, as this method is more sensitive to small variations compared to a direct extraction of strain values from peak positions at specific azimuths on the rings. Contrary to Rietveld analysis, an integration was performed only for sectors of 1° of the ring using fit2D software [13]. Thus, we obtained 360 diffractograms (2θ, intensity) for the 360 azimuths (δ) that constitute the ring. In order to extract stresses in each phase from these data, it is necessary to determine the 2θ position of one peak in each phase. We selected $\alpha^{\prime }\left(211\right)$, γ(200), and TiC(220), as they did not overlap with other peaks. Diffraction peaks were approximated by a Pearson VII function that allows the shape of our peaks to be reproduced and for their 2θ position, full width at half maximum (FWHM), intensity, shape factor, and background parameters to be obtained. This operation was repeated for the three phases for the 360 azimuths for each image collected during the thermal treatment.An automatic procedure was developed in Python to analyse the data (approximately 3 million peaks were analysed per phase extracted from 150 GB of raw data).

To account for uncertainties in the 2θ position due to the variations of the beam position over time, the average 2θ position for the opposite azimuths (δ and δ + 180°) was calculated. This step allowed us to free ourselves of the exact knowledge of the position of the centre of the ring [14].

**Figure 4:**
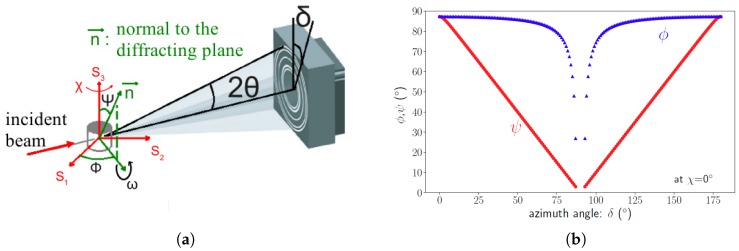
(**a**) Angles definition from setup configuration: (S1, S2, S3) is the sample reference system and (**b**) evolution of (Φ,Ψ) defining the direction normal to the diffracting plane {hkl}.

In order to apply the $sin^{2}\Psi$ method, it is necessary to convert our configuration to the classical (Φ, Ψ) configuration (Figure 4 presents a sketch of the setup and describes the angles). Indeed, each azimuth corresponds to a given pair of angles (Φ, Ψ) that can be determined by the following equations [15,16]:

(1) $$cos\Psi =cosc{\left(\left(1-\frac{sin^{2}\theta \;sin^{2}\delta }{1-cos^{2}c}\right)\left(1-\frac{sin^{2}\omega \;sin^{2}\delta }{1-cos^{2}c}\right)\right)}^{1/2}-\frac{sin\omega \;sin\theta \;sin^{2}\delta }{1-cos^{2}c},$$

where

(2) cosc=cosθcosω+sinθsinωcosδ,

(3) $$\Phi =\chi +arccos\left(\frac{sin(\theta -\omega )}{sin\Psi }\right),$$

with θ as the Bragg angle, χ as the sample rotation, and ω as the sample tilt. Thus, angle pairs (Φ, Ψ) were distributed as shown in Figure 4 versus their position on the ring (azimuth δ).

Finally, the $sin^{2}\Psi$ method can be applied. The deformation $\epsilon_{\Phi \Psi }$ in the direction normal to the diffracting plane defined by Φ and Ψ angles is expressed as a function of the components of the deformation tensor $\epsilon_{ij}$ in the sample reference system:

(4) $$\begin{array}{c} \epsilon_{\Phi \Psi }=ln\left(\frac{sin\theta_{0}}{sin\theta }\right)=\left[\left(\epsilon_{11}cos^{2}\Phi +\epsilon_{12}sin2\Phi +\epsilon_{22}sin^{2}\Phi \right)-\epsilon_{33}\right]sin^{2}\Psi +\\ \left(\epsilon_{13}cos\Phi +\epsilon_{23}sin\Phi \right)sin2\Psi +\epsilon_{33}. \end{array}$$

Introducing Hooke’s law, $\epsilon_{\Phi \Psi }$ can be expressed as a function of the components of the stress tensor:

(5) $$\epsilon_{\Phi \Psi }=ln\left(\frac{sin\theta_{0}}{sin\theta }\right)=\frac{1+\nu_{hkl}}{E_{hkl}}\left(\sigma_{\Phi }-\sigma_{33}\right)sin^{2}\Psi +\frac{1+\nu_{hkl}}{E_{hkl}},$$

with:

(6) $$\tau_{\Phi }sin2\Psi +\frac{1+\nu_{hkl}}{E_{hkl}}\sigma_{33}-\frac{\nu_{hkl}}{E_{hkl}}Tr\left(\bar{\bar{\sigma }}\right),$$

(7) $$\sigma_{\Phi }=\sigma_{11}cos^{2}\Phi +\sigma_{12}sin2\Phi +\sigma_{22}sin^{2}\Phi ,$$

(8) $$\tau_{\Phi }=\sigma_{13}cos\Phi +\sigma_{23}sin\Phi ,$$

(9) $$Tr\left(\bar{\bar{\sigma }}\right)=\sigma_{11}+\sigma_{22}+\sigma_{33}.$$

$E_{hkl}$ and $\nu_{hkl}$ are the X-ray elastic constants (Young’s modulus and Poisson’s ratio). $\theta_{0}$ is the stress-free diffraction angle for the diffracting plane. Then, $ln\left(\frac{1}{sin\theta }\right)=f\left(sin2\Psi \right)$ was plotted for each phase. For triaxial stress states, an ellipse is obtained (without texture) for a given Φ angle. The shear stresses are determined from the ellipse opening. The slope of the ellipse axis allows the stress difference $\sigma_{\Phi }-\sigma_{33}$ to be obtained, where $\sigma_{33}$ is the stress component along the sample axis. By selecting the rotation position corresponding to a specific orientation of the transverse section of the sample, we can extract the $\sigma_{11}$–$\sigma_{33}$ and $\sigma_{22}$–$\sigma_{33}$ stress differences from positions (0°, 180°) and (90°, 270°) versus temperature. These stress differences are the mean values for the considered phase.

Because the intercept (Equation (10)) of the $sin^{2}\Psi$ curve (at Ψ = 0) is related to $\epsilon_{33}$ [17], it is possible to determine all the strain components $\epsilon_{ii}$ and then determine the stress components $\sigma_{ii}$ if $\theta_{0}$ is known:

(10) $$Intercept=\epsilon_{33}+ln\left(\frac{1}{sin\theta_{0}}\right).$$

**Figure 5:**
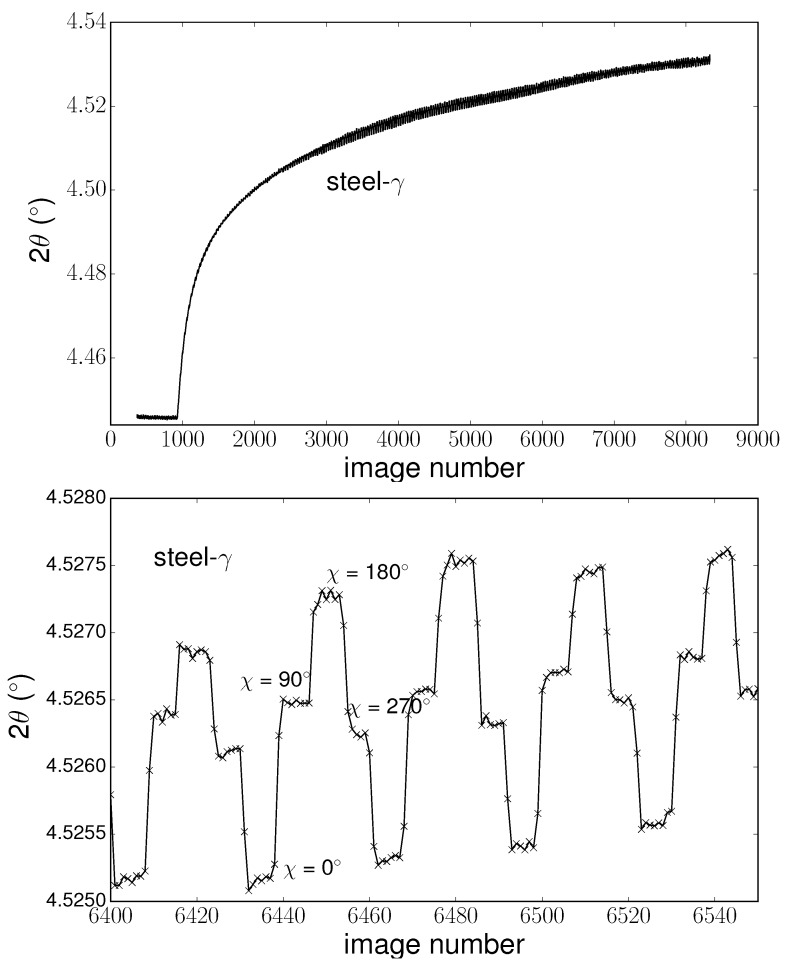
Evolution of peak position (γ (200)) over image number, and a zoomed view of image numbers 6400–6550.

Nevertheless, data on the intercept need to be corrected, as the values are sensitive to the sample’s tilt (as shown in Section 5.5). Due to the thermal expansion of the sample and its holder and also to the weight of the thermocouple, a tilt was identified during the sample rotation that resulted in a small variation of the peak position over time, correlated with sample rotation. In Figure 5, an example of the evolution of peak position over time is presented, in addition to a zoomed in view of a selected range of image numbers. We could identify the image where the sample position was fixed (i.e., rotation stopped), and thus where the sample orientation was known. As the tilt was reproducible over time, we averaged the intercept values corresponding to equivalent orientations and given temperature.

As already seen, to obtain the full strain and stress tensor, we need $\theta_{0}$ (i.e., the stress-free cell parameters of the phases and their evolution versus temperature). For steel-γ phase and TiC, we used the value of the cell parameters measured at high temperature assuming, that stresses were fully relaxed at 900 °C. For martensite values, we used values from the literature for a steel with composition close to the one in this study at room temperature [18]. The evolution of stress-free cell parameter versus temperature was calculated using the thermal expansion coefficient of phases, extracted from experimental studies for steel [9] or from the literature for TiC [19]. For our analysis, we used the isotropic elastic constants (Young’s modulus E and Poisson’s ratio ν). For the steel [20] and for the TiC reinforcements [19], the evolution of E and ν with temperature were taken from the literature as well as for TiC reinforcements [19]. Using macroscopic elastic constants we assume that the mechanical anisotropy of all phases can be neglected.

## 5. Results

### 5.1. Phase Transformation Kinetics

**Figure 6:**
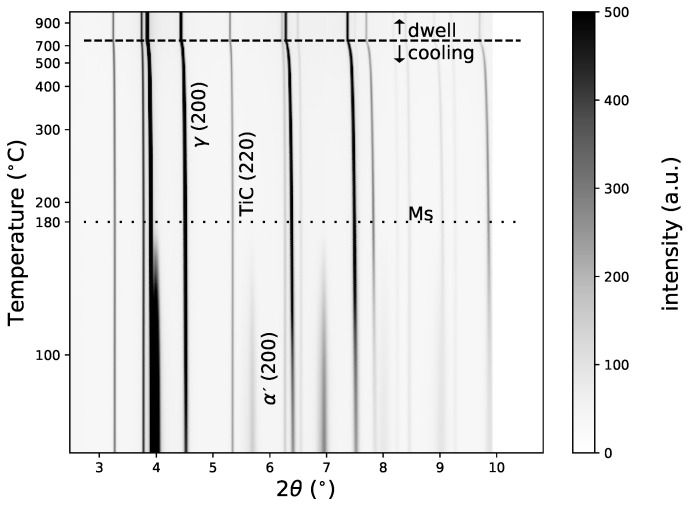
Evolution of diffractograms during cooling.

**Figure 7:**
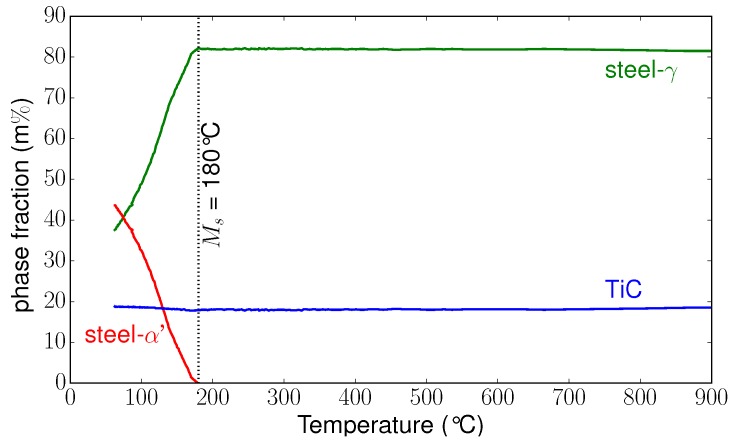
Phase fraction evolutions during cooling.

Figure 6 presents the evolution of the integrated 2D images versus temperature during the dwell and cooling periods. During cooling, we could identify the phase transformation of the matrix by the intensity decrease of the peak corresponding to the γ phase (face-centred cubic structure) and the increase of the $\alpha^{\prime }$ phase (tetragonal structure). In Figure 7, we present the evolutions of the phase fractions during the cooling from 900 °C to 50 °C, leading to the formation of martensite in the matrix (as mentioned previously in Section 2). At the beginning of the cooling, only austenite (γ) and TiC were present, with 81 m% and 19 m% respectively. During cooling, the amount of TiC remained constant. The martensitic transformation began at 180 °C. From 180 °C to 50 °C, we could follow the increase of martensite fraction and decrease of austenite fraction. At 50 °C, the microstructure was composed of 54% martensite and 46% retained austenite.

### 5.2. Evolution of Mean Cell Parameters

**Figure 8:**
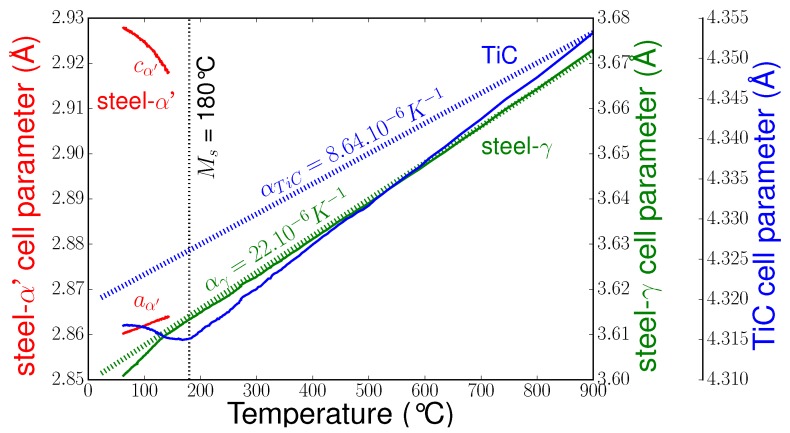
Cell parameter evolutions during cooling (stress-free thermal expansion coefficients of TiC and austenite are also shown).

From the beginning of cooling until the beginning of martensitic transformation, the cell parameters of the different phases decreased continuously without change of slope (Figure 8). From a previous analysis [21] of the apparent coefficient of thermal expansion (CTE), it was concluded that thermal stresses are generated in the phases during cooling. TiC was under a mean compression state since the CTE of austenite is about three times larger than that of TiC. Indeed, as shown in Figure 8, the apparent CTE of TiC was much higher than the stress-free CTE. Before the martensitic transformation, the mean stress state in austenite seemed very small.

As the martensitic transformation occurred, the TiC cell parameter increased, indicating a decrease of the mean compression in TiC. As the martensite content reached approximately 15%, the austenite cell parameter decreased, indicating an increase in compression. We could also observe an increase of the tetragonality (c/a ratio) of the martensite (the c parameter increased while the a parameter decreased linearly). Note that as the martensitic transformation began, peak intensities were very low (as can be seen in Figure 6), and thus accurate determination of cell parameters was not possible. Therefore, cell parameters for low fractions of martensite (below 10%) are not presented.

### 5.3. Full Width at Half Maximum

**Figure 9:**
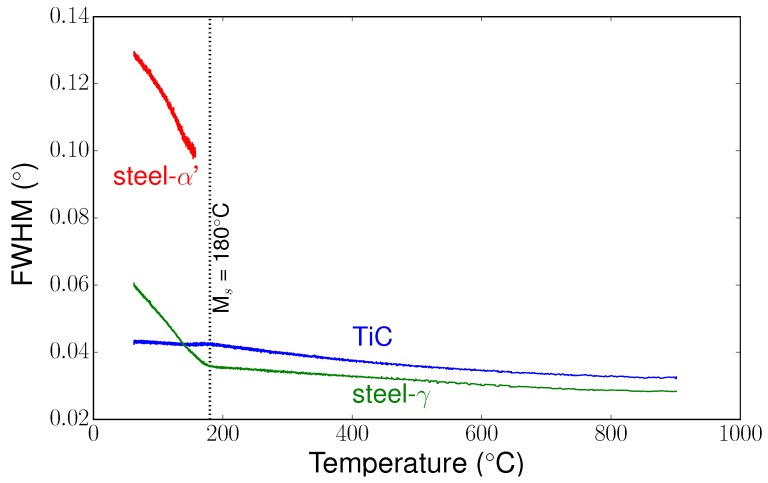
Evolution during cooling of the full width at half maximum (FWHM) for the different phases (steel-γ phase (200), TiC (220), and steel-$\alpha^{\prime }$ phase (211)) versus temperature.

In addition to the evolution of mean cell parameters, we also characterised the mean full width at half maximum (FWHM) evolutions for each phase during the heat treatment. From the integrated images, a fit was made using a Pearson VII function to extract the FWHM for each phase versus temperature. Figure 9 shows the evolution of the FWHM of selected peaks for the different phases present in the composite. Between 900 °C and the martensitic start temperature (Ms) temperature, the FWHM could be linked to heterogeneities of thermal stresses generated in the phases. At Ms, the FWHM for γ phase increased more sharply. A sharp increase was also observed for the $\alpha^{\prime }$ phase. As the martensitic transformation occurs without a change in chemical composition, these evolutions of FWHM can be mainly attributed to heterogeneous transformation stresses due to the deformation associated with martensitic transformation. Below Ms, the FWHM for TiC remained nearly constant, indicating low stress gradients in the particles.

### 5.4. Stress Evolutions

**Figure 10:**
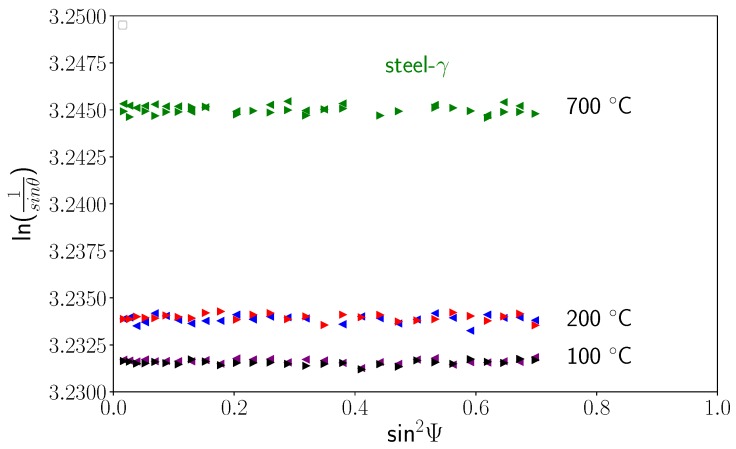

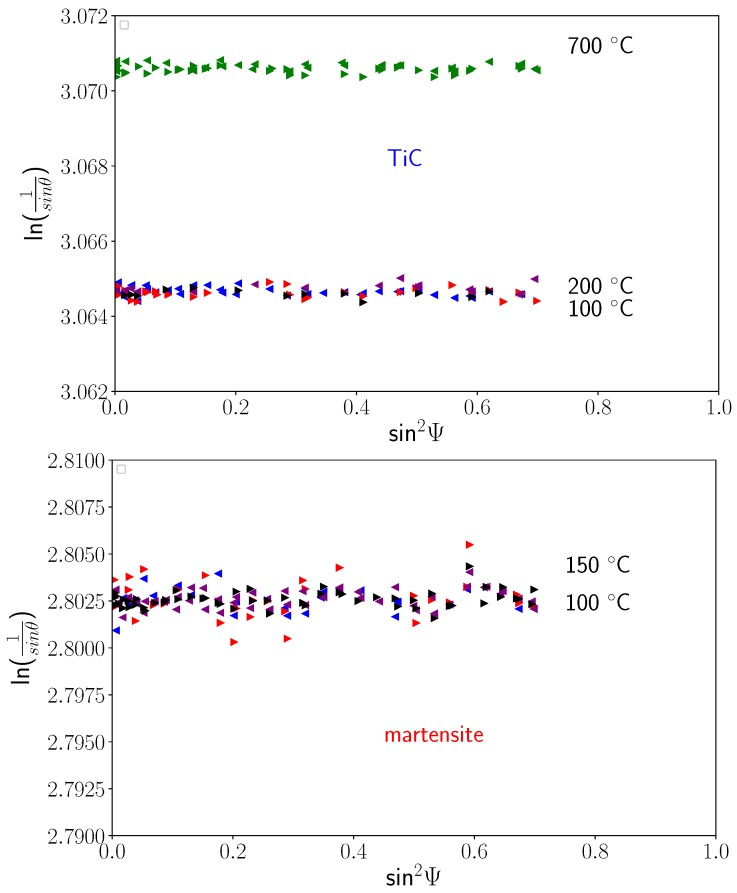
Evolution of the ${sin}^{2}\Psi$ curves at different temperatures for steel-γ phase, TiC, and steel-$\alpha^{\prime }$ phase.

**Figure 11:**
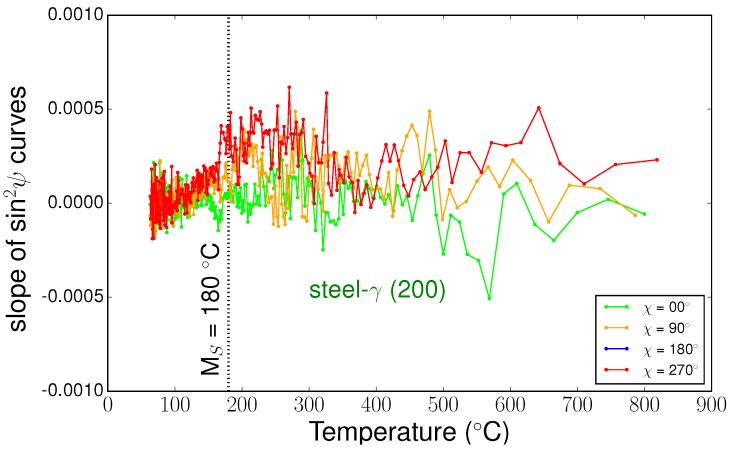

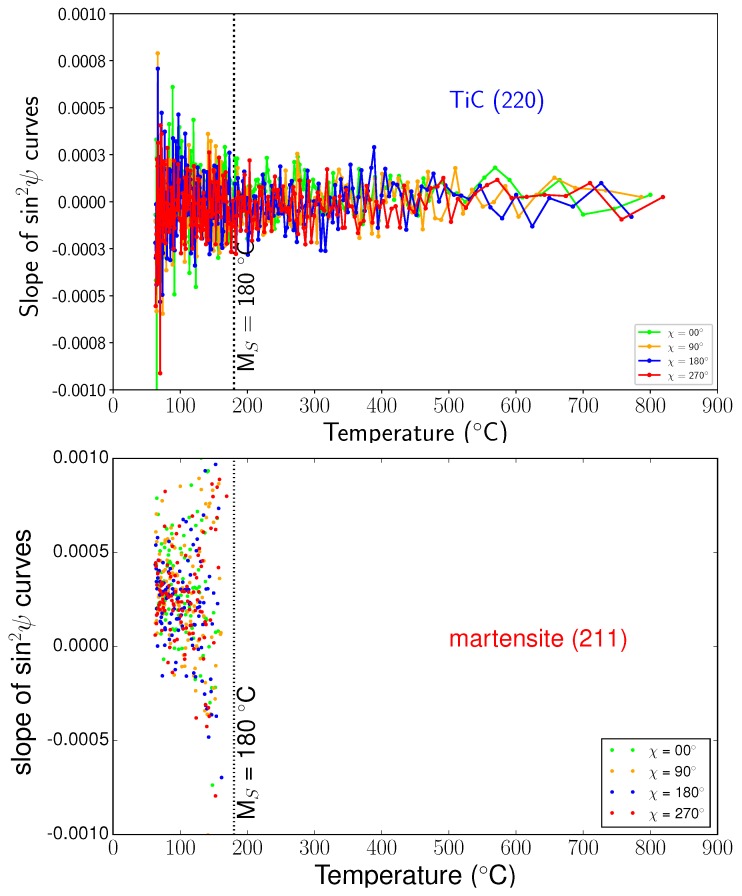
Evolution of the slopes of the ${sin}^{2}\Psi$ curve versus temperature for steel-γ phase, TiC, and steel-$\alpha^{\prime }$ phase.

**Figure 12:**
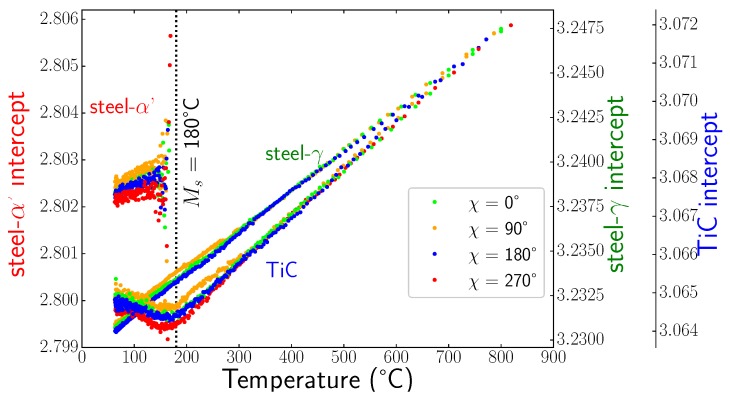
Evolution of the intercepts of the ${sin}^{2}\Psi$ curves versus temperature for different χ angle for steel-γ phase, TiC, and steel-$\alpha^{\prime }$ phase.

Using $sin^{2}\Psi$ method, we were able to determine all the components of the stress tensors in the different phases during cooling. Figure 10 shows some examples of $sin^{2}\Psi$ curves for the three phases at different temperatures. We can see that the curves were close to straight lines, meaning that shear stresses were negligible, and the slopes were very small. This is confirmed in Figure 11, which presents the evolutions of the slopes of $sin^{2}\Psi$ curves (i.e., the stress differences) versus temperature for the different phases. Thus, the mean stress states remained hydrostatic in all phases during cooling, even during the martensitic transformation. In the following, we only present the results for $\sigma_{ii}$ components, as the other components $\sigma_{ij,i\neq j}$ were negligible. Figure 12 shows the evolution of the intercept of the $sin^{2}\Psi$ curve versus temperature for the different phases. As mentioned previously, the intercept evolution was sensitive to the tilt of the sample during rotation. This effect was clearly seen for temperatures lower than 300 °C for TiC particles and austenite, where the different curves corresponded to different χ angles. In our approach, we corrected this tilt artefact by using average values of the intercepts.

**Figure 13:**
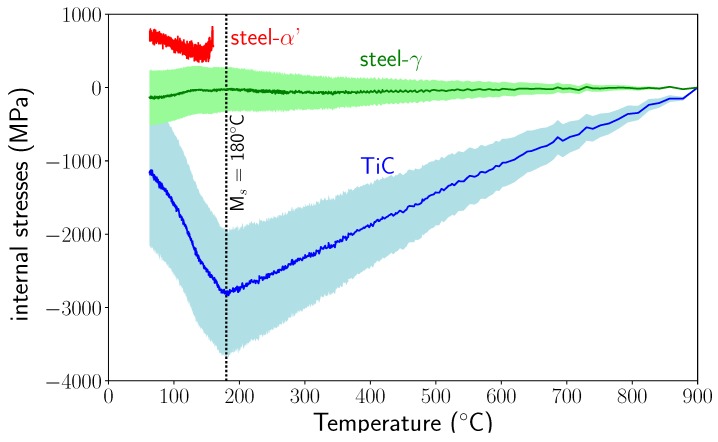
Internal stress evolutions during cooling (because the stress states are hydrostatic, only one component of the stress tensor is presented). Envelopes show the stress variations introduced by uncertainties in thermal expansion coefficients (see Section 5.5).

Finally, Figure 13 presents the determined stress evolutions during cooling from 900 °C to 50 °C.

Note the high level of compressive stress in TiC increasing from the high temperature until the appearance of martensite. The stress level in austenite was relatively low. The martensitic transformation induced a large decrease of the compressive stress in TiC and small compressive stresses in austenite. For martensite, the tensile stress first decreased and then increased as temperature decreased.

### 5.5. Discussion

From the experimental results, we can say that the calculated macroscopic stress for the composite (using the measured phase fractions) was not zero, even before the phase transformation. Note that stress values in the phases were highly dependent on the stress-free cell parameters and CTE, and to a lesser extent on X-ray elastic constants. In the following, we give an estimation of the possible effect of these parameters on the determined stress levels.

#### 5.5.1. Coefficient of Thermal Expansion

From a literature review on the coefficient of thermal expansion of TiC [19,22,23] and experimental results for steels, we estimated that the error on CTE is around (±1.10^−6^ K^−1^). In Figure 13, the envelopes present the variations in the stress levels for austenite and TiC, including these experimental uncertainties (for martensite, the stress variations are not shown as they would overlap the stress scattering). We can see that the variation in stress level can be very large. There were even variations large enough to find values of CTE for which the macroscopic stresses were zero.

#### 5.5.2. Stress-Free Parameters

For steel and TiC, the stress-free parameters were estimated by Rietveld refinement on the diffractograms at the end of the dwell at 900 °C, assuming stress relaxation at 900 °C. We compared the value of the cell parameters for a dwell at 950 °C. The Rietveld analysis showed a variation of the cell parameters close to the thermal expansion between 900 and 950 °C, thus justifying our assumption.

Another difficulty came from the fact that values of stress-free parameters of martensite are very difficult to obtain since local stresses are generally generated as martensite forms. As described above, in our approach, we used values from the PDF-4 database, which are close to the ones determined by Roberts [18]. This means that the level of stress in martensite is unknown. To estimate the stress level in martensite, the criterion of macroscopic stress equal to zero in the composite could be used.

#### 5.5.3. Macroscopic Elastic Constants

Different values for the Young’s Modulus and Poisson’s ratio for TiC can be found in the literature [19,22,24,25], depending on the chemical composition of the TiC (nitrogen can be substituted for carbon and the stoichiometry of the structure is not perfect). Values from Wall et al. [19] used in our study are close to the average values of the different studies. If we take the extreme values found in the literature into account, the maximum level of compressive stress reached before M_*S*_ temperature could vary by about 250 MPa.

### 5.6. Micromechanical Modelling

In order to better understand the experimental results, 3D finite element micromechanical modelling (Code Zebulon [26]) was performed to calculate the internal stress evolutions during cooling.

#### 5.6.1. Description of the Model

**Figure 14:**
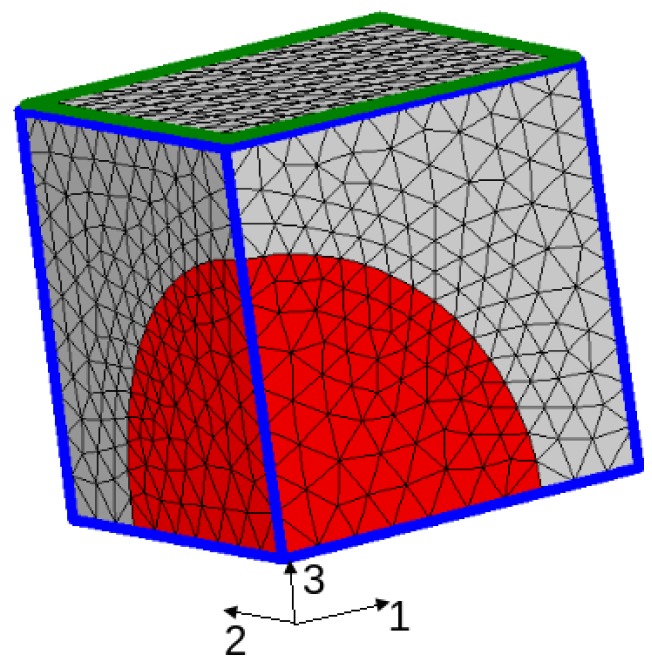
Unit cell used for the 3D finite element micromechanical modelling.

Calculations were performed for a 3D simplified microstructure: a periodic distribution of spherical TiC particles (representing 25% volume fraction) embedded in a steel matrix. Due to symmetry, only one eighth of the unit cell needed to be meshed (see Figure 14). Periodicity was imposed through the boundary conditions.

The thermal treatment was the same as that imposed during the experiments: cooling of the composite was simulated from 900 °C to room temperature by imposing cooling in accordance with Figure 2. Phases were assumed to be fully relaxed at 900 °C. Martensitic transformation was described by a Koistinen–Marburger law [27], with parameters determined from experimental results.

(11) $$y_{m}=\left(1-exp^{-\beta (Ms-T)}\right),$$

where $y_{m}$ is the martensitic mass fraction, Ms is the martensitic start temperature (Ms = 180 °C), and β is a coefficient equal to 0.014 °C^−1^. For the actual model, only two phases were taken into account: the matrix and the particles.

The model considers a a thermo-elasto-visco-plastic behaviour law of the matrix including the phase transformation deformations (volume change and transformation plasticity). This behaviour law derives from previous works on the prediction of internal stresses in metallic alloys [28,29]. This law is written in incremental form by:

(12) $$d\epsilon_{ij}^{t}=d\epsilon_{ij}^{e}+d\epsilon_{ij}^{p}+d\epsilon_{ij}^{th}+d\epsilon_{ij}^{tr}+d\epsilon_{ij}^{pt},$$

with:

d$\epsilon_{ij}^{e}$: incremental elastic strain related to stress increment by Hooke’s law with temperature-dependent Young’s modulus and Poisson’s ratio.
d$\epsilon_{ij}^{p}$: incremental visco-plastic strain at high temperature and plastic strain at lower temperatures.

These components are calculated using the classical plasticity theory with Von Mises criterion and isotropic hardening.

For each phase *k*, the yield stress $\sigma_{k}$ is determined by:

(13) $$\sigma_{k}=\sigma_{0k}+H_{k}\epsilon_{vp}^{n_{k}}+K_{k}{\dot{\epsilon }}_{vp}^{m_{k}},$$

where $\sigma_{0k}$ is the threshold stress, $H_{k}\epsilon_{vp}^{n_{k}}$ is the hardening by plastic strain, and $K_{k}{\dot{\epsilon }}_{vp}^{m_{k}}$ is the viscous stress. H_*k*_, n_*k*_, K_*k*_, and m_*k*_ are temperature-dependent coefficients determined experimentally for each phase.

d$\epsilon_{ij}^{th}$: incremental thermal strain

(14) $$\epsilon_{ij}^{th}=\sum y_{k}\int \alpha_{k}\left(T\right)dT,$$

where $\alpha_{k}$ is the temperature-dependent coefficient of thermal expansion of phase *k* and y_*k*_ is the volume fraction of phase *k*.

The strain increments due to phase transformation are:

d$\epsilon_{ij}^{tr}$: incremental strain due to volume change
  (15) $$\epsilon_{ij}^{tr}=y_{k}\sum \epsilon_{k,0\;{}^{\circ }C}^{tr},$$
d$\epsilon_{ij}^{pt}$: incremental strain due to transformation plasticity
  (16) $$d\epsilon_{ij}^{pt}=\frac{3}{2}K_{k}f^{\prime }\left(y_{k}\right)dy_{k}s_{ij},$$
  where $s_{ij}$ are the components of the deviatoric stress tensor, and $K_{k}$ is an experimentally determined coefficient. For martensitic transformation $f\left(y_{m}\right)=\left(2-y_{m}\right)y_{m}$.

All data concerning the steel matrix (austenite and martensite) were extracted from previous studies done in our laboratory. For TiC reinforcements, a thermo-elastic behaviour was considered using data from the literature [19].

#### 5.6.2. Calculated Results

**Figure 15:**
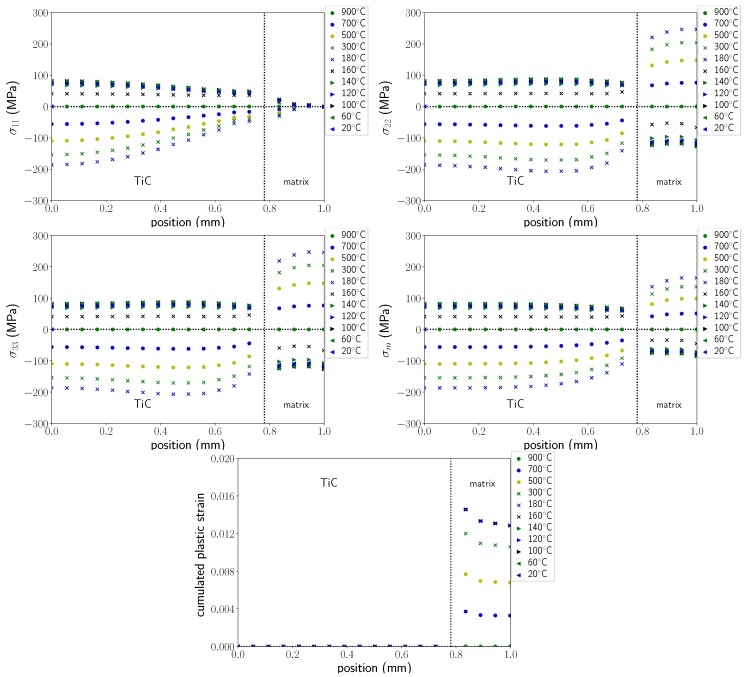
Stresses and cumulated equivalent plastic strain profiles along axis 1 for different temperatures.

Calculations allow the analysis of stress and deformation gradients in the particles and in the matrix during the entire cooling process. Figure 15 presents the stress profiles (components $\sigma_{11}$, $\sigma_{22}$, $\sigma_{33}$ and $\sigma_{m}$ (hydrostatic stress)) along axis 1 of the cell (see Figure 14) for temperatures ranging from 900 °C to 20 °C.

The profiles of the cumulated equivalent plastic strain in the matrix are represented in Figure 15. Before the martensitic transformation, stress profiles show compressive stresses in the particle, as expected from the differences in thermal expansion coefficients. These stresses increased as temperature decreased, and reached −200 MPa at 180 °C in the centre of the particle. It can be noticed that stress gradients existed in the particle with lower stresses close to the interface, particularly for $\sigma_{11}$, and that the stress state was not hydrostatic as would be predicted by Eshelby’s model [30]. This is mainly related to the interactions between the particles, as the volume fraction was relatively high (25%).

In the matrix, $\sigma_{11}$ was small. $\sigma_{22}$ and $\sigma_{33}$ increased in tension as temperature decreased, and showed relatively small gradients. Hydrostatic stress was approximately 160 MPa at the boundary of the cell at 180 °C. Viscoplastic strains developed in the matrix (austenite) from the beginning of cooling and reached a maximum value (about 1.5%) just before the martensitic transformation. As the martensitic transformation occurred, a large stress relaxation occurred in the matrix as well as in the particle that led to a stress reversal—stresses in the matrix and in the particle became respectively compressive and tensile. This stress relaxation was due to the volumetric variation associated with martensitic transformation. Due to this stress relaxation, no more plastic strains developed in the matrix, but as the transformation progressed, transformation plasticity strains (not shown here) were generated. Finally, the residual stress state was tensile in the particle and compressive in the matrix.

#### 5.6.3. Comparison with Experimental Results and Discussion

**Figure 16:**
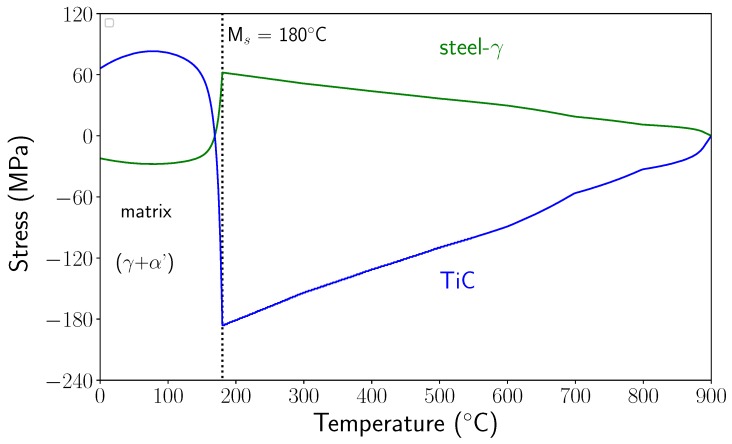
Evolution of the mean calculated stresses in the reinforcements and in the matrix during cooling.

In order to compare the micromechanical results with the experimental ones, the mean values of the stresses were calculated in the matrix and in the reinforcement during the entire cooling process. The mean values were calculated from the stress tensor components in each finite element weighted by the element volume. The evolution of the mean stresses in the matrix and in TiC particles during cooling are presented in Figure 16.

Between 900 °C and Ms temperature, the stresses increased in tension and compression for the matrix (which was austenitic) and in compression in the TiC particles. At Ms, they reached +62 MPa in the matrix and −185 MPa in the particle. As noted above, martensitic transformation induced a large stress relaxation, particularly in the reinforcements. Residual stresses were tensile in TiC and compressive in the matrix, with relatively low levels. These evolutions were in accordance with the evolutions observed experimentally, but the stress levels were very different. In particular, in TiC, just before the matrix phase transformation, the calculated stress level was 10 times smaller than the experimentally determined value.

Note that experimental stress values in TiC particles were much closer to those calculated considering a thermoelastic behaviour of the matrix. Indeed, a calculation performed considering a thermoelastic behaviour of the matrix led to a stress level in the TiC particles just before Ms of about −1600 MPa. Thus, it seems that our calculation overestimated the (visco)plastic deformations in the austenite during cooling before the martensitic transformation. This assumption is reinforced by the fact that only small variations of the FWHM of austenite were observed before the martensitic transformation (see Section 5.3). Thus, questions arise about the accommodation of the thermal stresses generated during cooling (before the transformation). One important point is that the real microstructure was very different from the modelled one. As mentioned previously, the actual microstructure was very heterogeneous and there are reinforced zones where the volume fraction of particles is very high (50% or even more) and zones without particles that could lead to different plastic strain development. In addition, in the reinforced area, interparticle distances were very small (on the order of 1 µm), and would probably lead to less plastic accommodation, as simulated presently.

Except for the questions addressed above, it can be said that the main contribution of our study is to reveal the strong influence of the phase transformation of the matrix on the internal stress evolutions in the composite material. Indeed, in the literature, a number of studies deal with phase transformations and microstructural changes during the processing of composite materials or during heat treatment, either phase transformation of the reinforcements [6,31,32,33] or within the matrix [34,35], and their consequences on the mechanical behaviour. However, a thorough analysis of the role of the phase transformation in the internal and residual stresses and their in situ quantification has not yet been performed. In previous work on different nonreinforced steels [36], it was shown experimentally through the evolution of cell parameters that martensitic transformation induces compressive stresses in the austenite, once about 15% martensite has been formed. This was an unexpected behaviour, since tensile stresses were expected in the austenite.

Indeed, the free strain associated with the formation of a martensite plate has two contributions: a volumic strain and a shear strain. The order of magnitude of the shear strain is 0.2. The shear strain may be accommodated by the formation of self-accommodating plates, and/or by elastic and plastic deformation. The volumic strain is positive (its order of magnitude for steels varies from 1.5% to 3%), and is accommodated by elastic and plastic deformations. Considering Eshelby’s model, the formation of a martensite plate will lead to a mean compressive stress in the martensite, and a mean tensile stress in the infinite medium of austenite. The building up of compression stresses in the parent austenite is still complex to understand, but we explain it by the increasing interactions between the martensite plates as transformation progresses. An increase of FWHM in austenite during the phase transformation was also observed for martensite amounts larger than 15% and was related to elastic strain heterogeneities.

Here, in the case of our reinforced steel, through the cell parameter evolutions (Figure 8), we also revealed compressive stresses in the austenite once the amount of martensite reached 15%. FWHM evolutions also revealed elastic heterogeneities in austenite as well as in martensite resulting from the accommodation of the transformation strain. However, the very new feature is that the martensitic transformation of the matrix induced an unexpected high-stress relaxation in the particles, and this can only be explained by the micromechanical approach that considers the volumic variation associated with the martensitic transformation. Consequently, the phase transformation of the matrix also had a strong influence on the residual stress states after cooling in the composite material. From the micromechanical approach, it was also shown that the accommodation mechanisms of the thermal and transformation stresses (elasticity, plasticity, etc.) were determinant on the level of internal and residual stresses, and in the future deeper studies on that point are necessary: determination of local strains, stresses, and dislocation densities by synchrotron (Laue microdiffraction) and high-resolution TEM.

## 6. Conclusions

We developed an original experimental device and a methodology for robust and rapid in situ stress analysis starting from a very large number of 2D images (i.e., Debye–Scherrer rings) obtained by synchrotron X-ray diffraction experiments during heat treatment. Indeed, the device allows control of not only heating and cooling, but also the rotation of the sample in order to obtain data in all necessary directions to extract the informations on the evolution of strains and stresses. As far as we know, it is the first time such experiments have been carried out. From the present experiment performed during the heat treatment of a steel matrix composite reinforced by TiC particles, we determined the evolutions of the full-stress tensor in the different phases: austenite, martensite and TiC. We showed that the mean stresses in the phases remained hydrostatic during the entire cooling process. Large stresses were generated in the particles due to the differences in thermal expansion coefficient with austenite. The role of the martensitic transformation of the matrix on the stress states in the particles was clearly demonstrated. It was shown that martensitic transformation led to a high stress relaxation in the TiC particles and to a lesser extent in the matrix.

The 3D finite element micromechanical model allows detailed analysis of the stress gradients in the matrix and in the particles during the entire cooling process. It was shown that internal stresses resulting from the thermal expansion mismatch between the austenite and TiC particles may be high enough to lead to plastic accommodation in the matrix, resulting in relatively low stress levels in the particles. It was demonstrated that the martensitic transformation, due to the volumetric variation and transformation plasticity, led to a high stress relaxation in the particles and in the matrix. Nevertheless, the calculated mean values of the stresses in the different phases, even if the evolutions were similar, showed large discrepancy with the experimental results, especially in the particles. These discrepancies were related on one hand to experimental uncertainties in stress levels due to uncertainties in thermal expansion coefficients and stress-free cell parameters. On the other hand, the present micromechanical calculations on a simplified microstructure probably overestimated the plastic accommodation in the matrix. Work is on course for taking the heterogeneity of the real microstructure into account by performing micromechanical simulations on SEM images of the microstructure.

The results obtained in this paper also address the wider issue of better mastering (i.e., tailoring) the mechanical performance of MMCs. Particularly, the understanding of damage mechanisms (fracture of particles, interface decohesion, etc.) and their modelling needs to consider not only microstructural features, but also the mechanical states of the phases inherited from materials processing (internal stresses, plastic strains, etc.), which is often neglected. For example, the studies on steel–TiC composites obtained by SLM (selective laser melting) [5,37] pointed out the occurrence of cracks resulting from the high internal stresses induced on one hand by the solidification shrinkage and on the other hand by the rapid heating and cooling in the solid state. Experimental knowledge of internal stress evolutions as well as their numerical prediction in order to better control and optimise them is thus a key issue for different processing routes of composite materials and particularly additive manufacturing processes.

## Figures and Tables

**Table 1:** Chemical composition of the steel matrix 32CrMoV13 (data from Mecachrome).

C	Cr	Mo	Mn	Si	V	Ni	N
0.312	3.831	0.721	0.434	0.583	0.136	0.067	152 ppm
